# Validity and reliability of Eforto®, a system to (self-)monitor grip strength and muscle fatigability in older persons

**DOI:** 10.1007/s40520-023-02365-3

**Published:** 2023-03-10

**Authors:** Liza De Dobbeleer, Myrthe Manouk Swart, Merle Anne Joëlle Geerds, Remco Johan Baggen, Anne-Jet Sophie Jansen, Rudi Tielemans, Hugo Silva, Siddhartha Lieten, Kurt Barbé, Geeske Peeters, Miriam Marie Rose Vollenbroek-Hutten, René Johannes Franciscus Melis, Ivan Bautmans

**Affiliations:** 1 Gerontology Department, Faculty of Medicine and Pharmacy, Vrije Universiteit Brussel, Brussels, Belgium; 2 Frailty in Ageing Research (FRIA) Group, Faculty of Medicine and Pharmacy, Vrije Universiteit Brussel, Laarbeeklaan 103, B-1090 Brussels, Belgium; 3 Department of Geriatrics, Universitair Ziekenhuis Brussel, Brussels, Belgium; 4 Department of Geriatrics, Radboud Institute for Health Sciences, Radboud University Medical Center, Nijmegen, The Netherlands; 5 Department of Trauma Surgery, Ziekenhuisgroep Twente, Almelo, The Netherlands; 6 UniWeb, Meise, Belgium; 7 Instituto de Telecomunicações, Instituto Superior Técnico, Lisbon, Portugal; 8 Department Public Health (GEWE), Vrije Universiteit Brussel, Brussels, Belgium; 9 Research Group Biostatistics and Medical Informatics (BISI), Vrije Universiteit Brussel, Brussels, Belgium; 10 ZGT Academy, Ziekenhuisgroep Twente, Almelo, The Netherlands; 11 SOMT University of Physiotherapy, Softwareweg 5, 3821 BN Amersfoort, The Netherlands

**Keywords:** Intrinsic capacity, Muscle fatigability, Self-monitoring, Prevention, Frailty

## Abstract

**Introduction:**

We developed Eforto®, an innovative system for (self-)monitoring of grip strength (GS) and muscle fatigability (Fatigue Resistance (FR = time until GS decreased to 50% of maximum during sustained contraction) and grip work (GW = area under the strength-time curve)). The Eforto® system consists of a rubber bulb that is wirelessly connected to a smartphone-based application, and a telemonitoring platform. The aim was to evaluate the validity and reliability of Eforto® to measure muscle fatigability.

**Methods:**

Community-dwelling older persons (*n* = 61), geriatric inpatients (*n* = 26) and hip fracture patients (*n* = 25) were evaluated for GS and muscle fatigability. In community dwellers fatigability was tested twice in the clinic (once with Eforto®, once with Martin Vigorimeter (MV), standard analog handgrip system) and for six consecutive days as a self-assessment at home with Eforto®. In hospitalized participants, fatigability was tested twice using Eforto®, once by a researcher and once by a health professional.

**Results:**

Criterion validity was supported by good to excellent correlations between Eforto® and MV for GS (*r* = 0.95) and muscle fatigability (FR *r* = 0.81 and GW *r* = 0.73), and no significant differences in measurements between both systems. Inter-rater and intra-rater reliability for GW were moderate to excellent (intra-class correlation: 0.59–0.94). The standard error of measurement for GW was small for geriatric inpatients and hip fracture patients (224.5 and 386.5 kPa*s) and higher for community-dwellers (661.5 kPa*s).

**Discussion/conclusion:**

We established the criterion validity and reliability of Eforto® in older community-dwelling persons and hospitalized patients, supporting the implementation of Eforto® for (self-)monitoring of muscle fatigability.

**Supplementary Information:**

The online version contains supplementary material available at 10.1007/s40520-023-02365-3.

## Introduction

**Fig. 1:**
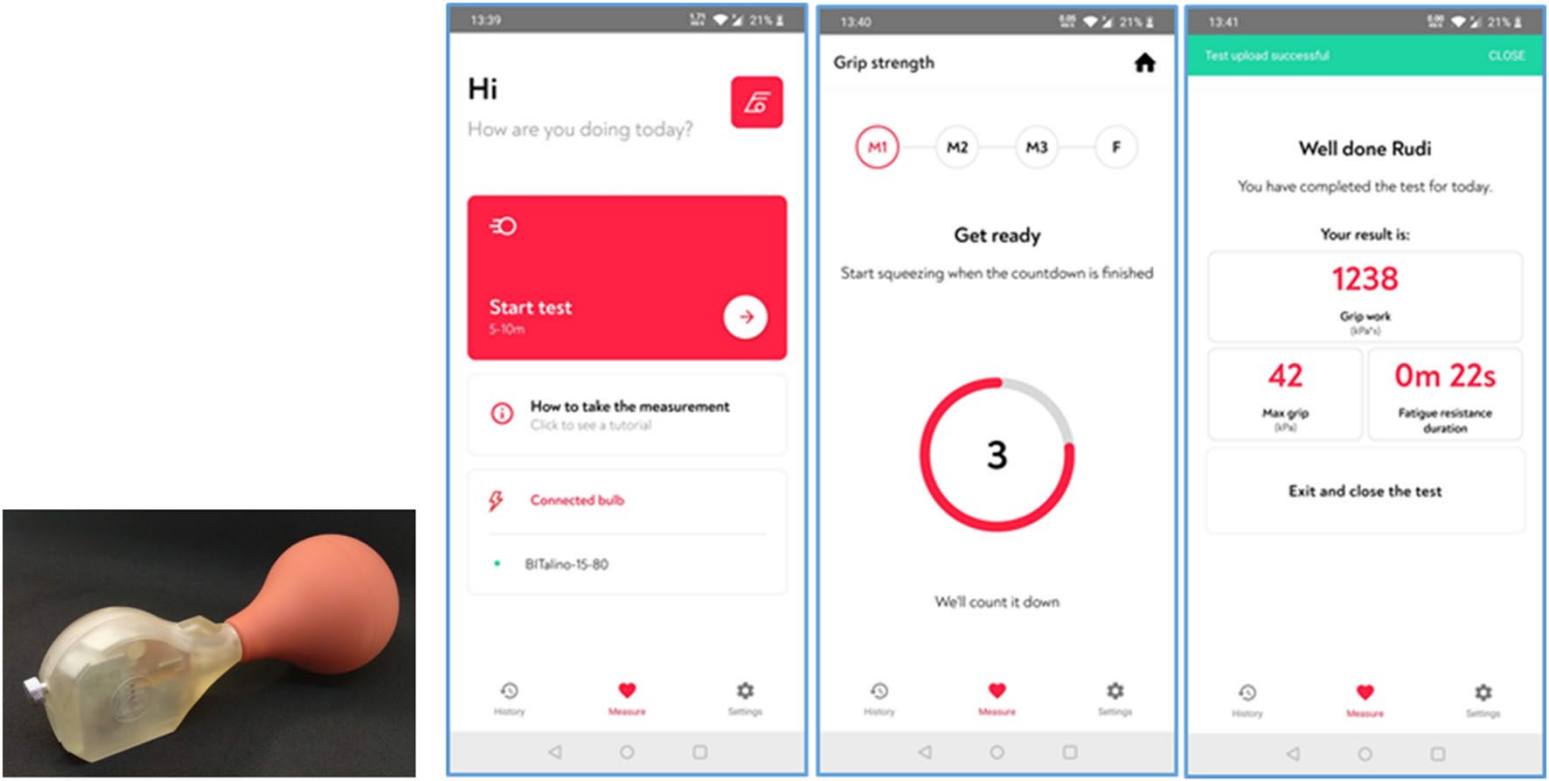
Eforto® device. From left to right: Eforto® device; start screen; time indicators for starting a self-test; overview of the test results

Loss of intrinsic capacity, the composite of all the physical and mental capacities that an individual can draw on [1], is a common health condition associated with ageing [1, 2], which leads to a loss of independence. Intrinsic capacity comprises all the mental and physical capacities that an individual can rely on. The intrinsic capacity level is affected by several factors such as the presence of diseases, injuries and age-related changes [3]. Various hypotheses on the aging process have been proposed, including genomic instability, epigenetic and metabolic disarray, oxidative stress, DNA damage, telomere shortening, inflammation, apoptosis, lipotoxicity, and mitochondrial modifications. Mitochondrial modifications have been extensively studied in aging conditions since they play an important role in different cellular pathways such as oxidative stress regulation, adenosine triphosphate biogenesis, mitophagy, and apoptosis [4–6]. These ageing processes affect muscle composition and function. Vice versa, muscle function may be a sensitive marker of progression of the ageing process and thus intrinsic capacity. The rapid increase in prevalence of older adults with compromised intrinsic capacity puts significant pressure on social and health care systems [7]. Solutions for early-onset detection and prevention of decreasing intrinsic capacity are currently unavailable. Existing technologies focus primarily on the detection of late-stage symptoms like frailty and dependency. Their assessment requires professional involvement, which makes them less suitable for long-term monitoring. Early identification and monitoring of changes in intrinsic capacity are key to better prevention and health management. Based on this need, we constructed a prototype smartphone-based system to assess muscle reserve recruitment capacity in persons with or without frailty, acting as an early warning system for the decline in intrinsic capacity. The Eforto® system (shown in Fig. 1) is designed to monitor muscle fatigability (fatigue resistance (FR) and grip work (GW)) as an ecological and dynamic marker of a person’s physical reserve capacity and resilience. The system facilitates (self-)assessment of muscle fatigability by measuring the maximal grip strength (GS_max_) an individual can apply and sustain until their GS drops to 50% of its maximum (i.e., FR-test [8–10]). Test results can be consulted remotely through a secured web-based platform.

Protocols for the measurement of FR and GW were originally developed and validated using the Martin Vigorimeter (MV, KLS Martin Group, Germany), which is a classic analog pneumatic handgrip system [8, 9]. Pneumatic systems, such as rubber bulbs, are better tools to measure muscle fatigability and more valid to discriminate between subjects of varying degrees of frailty compared with clamp-based instruments such as the Jamar dynamometer [11, 12]. Measurements with MV require involvement of trained professionals and is therefore less suitable for remote monitoring than Eforto®, which allows self-assessments with application support.

The aims of the present study were: (1) to assess the criterion validity of Eforto® against the analog MV measurement as a reference system in measuring muscle fatigability, and (2) to investigate the inter-rater and intra-rater reliability of muscle fatigability measured with Eforto® in community-dwelling older persons, hospitalized geriatric patients and older patients with hip fracture.

## Materials and methods

### Study sample

One hundred and twelve older persons from three ongoing studies were enrolled (Table 1): 61 community-dwellers aged 80+ years participating to the BrUssels sTudy on The Early pRedictors of FraiLtY (BUTTERFLY [13, 14], Vrije Universiteit Brussel & Universitair Ziekenhuis Brussel, Belgium), 26 hospitalized geriatric patients participating in the Bedside Resilience Registry (BRR, Radboud University Medical Center, The Netherlands) and 25 patients with hip fracture participating in the RESilience Hip fracture PatiEnts study (RESHAPE, Ziekenhuis Groep Twente, The Netherlands). Subjects consecutively enrolled in the study between August 2020 and November 2020 (inclusive). The study was conducted ethically in accordance with the World Medical Association Declaration of Helsinki. Following the COSMIN consensus guidelines for psychometric studies [15] we included a minimum of 50 participants per context for the psychometric evaluation for sufficient statistical power (patients are considered as 1 group here).

**Table 1:** Overview of test sample and protocol

Context	Site	Aim/study protocol	Participants’ characteristics
Community-dwelling older persons	VUB + UZB	Criterion validity: Muscle fatigability measured with analog vs Eforto® (professional mode), in random order, at the same day in hospital, at least 1 h interval, the same researcher for both test (*n* = 4) (exception: in 4 cases the researcher during test 1 was different from test 2)	*N* = 61 (34 women, 27 men) ⇒ 5 cases were excluded (2 women and 3 men) Mean age = 85.6 ± 3.3 years Living situation: *n* = 61 at home Medication: *n* = 3.2 ± 2.4 Mean Charlson’s Comorbidity Index = 5.25 ± 1.14
		Intra-rater reliability: Muscle fatigability measured with Eforto® (self-test mode), 7 days at home, once a day	*N* = 30 (15 women, 15 men) ⇒ 2 cases were excluded (1 woman and 1 man) Mean age = 86.3 ± 3.0 years Living situation: *n* = 30 at home Medication: *n* = 3.1 ± 2.0 Mean Charlson’s Comorbidity Index = 5.00 ± 0.79
		Inter-rater reliability: Muscle fatigability measured once in hospital by researcher (*n* = 2) with Eforto® (professional mode), 1 day later by participant at home (self-test mode)	*N* = 30 (15 women, 15 men) ⇒ 1 case was exluded (man) Mean age = 86.3 ± 3.0 years Living situation: *n* = 30 at home Medication: *n* = 3.1 ± 2.0 Mean Charlson’s Comorbidity Index = 5.00 ± 0.79
Hospitalized geriatric patients	Radboudumc	Inter-rater reliability: Muscle fatigability measured with Eforto® (professional mode), researcher (*n* = 2) vs health professional (*n* = 3), in random order dependent of workload/schedule, at the same day in hospital, at least 1 h interval (exception: 1 retest within 50 min)	*N* = 26 (16 women, 10 men) ⇒ 1 case was excluded (woman) Mean age = 84.1 ± 6.4 years Living situation: *n* = 23 at home, *n* = 2 in a nursing home/residential care centre/revalidation centre, *n* = 1 other Mean Charlson’s Comorbidity Index = 5.35 ± 1.3
Patients with hip fracture	ZGT	Inter-rater reliability: Muscle fatigability measured with Eforto® (professional mode), researcher (*n* = 1) vs health professional (*n* = 1), in random order, at the same day in hospital, at least 1 h interval	*N* = 25 (18 women, 7 men) ⇒ 2 cases were excluded (2 women) Mean age = 81.9 ± 7.0 years Living situation: *n* = 24 at home, *n* = 1 in a nursing home/residential care centre/revalidation centre Mean Charlson’s Comorbidity Index = 4.96 ± 1.57

*VUB* Vrije Universiteit Brussel (Belgium), *UZB* Universitair Ziekenhuis Brussel (Belgium), *Radboudumc* Radboud University Medical Center (The Netherlands), *ZGT* Ziekenhuisgroep Twente (The Netherlands), *GS*_*max*_ maximal grip strength, *FR* fatigue resistance, *GW*_*estimated*_ grip work estimated (Eq. 1), *GW*_*measured*_ grip work measured (Eq. 2), *ICC* intra class correlation coefficient, *SEM*_*agreement*_ standard error of measurement, *SDC* smallest detectable change, *SEP* systematic error proportion, *REP* residual error proportion

### In- and exclusion criteria

#### a.Community-dwelling older persons

Older volunteers were recruited among participants of the BrUssels sTudy on The Early pRedictors of FraiLtY (BUTTERFLY). Subjects were eligible if they were aged 80 and over, could walk independently, lived independently, if they were mentally fit (MMSE > 23/30), and not frail according to the Groningen Frailty Indicator (GFI < 4/15) [16], Rockwood Frailty Index (RFI < 0.25/10) [17], and/or the adapted Fried Frailty Index (FFI < 3/4) [18]; (exhaustion, weight loss, gait speed, and grip strength). Participants were excluded if they underwent surgery or any radiotherapy or chemotherapy during the past six months. Participants with CRP > 10 mg/l were excluded, as this refers to an acute inflammatory state and not to a chronic low-grade inflammatory profile [19]. The Eforto® assessments were added to the study protocol by means of an amendment, which was approved by the Medical Ethics Committee of the Universitair Ziekenhuis Brussel (B.U.N. 143,201,424,976). All participants provided written informed consent. All assessments took place at the Vrije Universiteit Brussel and the Universitair Ziekenhuis Brussel (Brussels, Belgium).

#### b.Hospitalized geriatric patients

Geriatric patients were consecutively recruited among participants of the Bedside Resilience Registry (BRR). All new admissions to the geriatrics ward meeting the in- and exclusion criteria (65+ years, expected hospital stay of more than two days, life expectancy of more than 14 days, speaking and understanding Dutch, no contact isolation, no severe cognitive impairment, no physical impairments for GS measurements) were eligible for the BRR study and the current sub study. The study was reviewed by the research ethics committee of the Radboud university medical center (file 2018-4973). It did not fall within the remit of the Medical Research Involving Human Subjects Act (WMO). The ethics committee approved the study based on the Dutch Code of conduct for health research, the Dutch Code of conduct for responsible use, the Dutch Personal Data Protection Act, and the Medical Treatment Agreement Act. All participants provided written informed consent. All assessments took place at Radboud University Medical Center (Nijmegen, The Netherlands).

#### c. Patients with hip fracture

Old patients with hip fracture were recruited for the RESilience Hip fracture PatiEnts study (RESHAPE) in the multidisciplinary Centre for Geriatric Traumatology at the Department of Trauma Surgery at Ziekenhuisgroep Twente Almelo-Hengelo. Participants meeting the in- and exclusion criteria (age > 70 years, surgically treated for a hip fracture, no total hip replacement, no pathological or periprosthetic fracture, no severe cognitive impairment, no terminal illness with life expectancy < 14 days and no physical impairments for grip strength measurements) were eligible for the RESHAPE study. The study was reviewed by the Local ethics committee Almelo (file ZGT20-42) and Medical Research Ethics Committee United, Nieuwegein (file W20.195). It did not fall within the remit of the Medical Research Involving Human Subjects Act (WMO). All participants provided written informed consent. All assessments took place at the Department of Trauma Surgery at Ziekenhuisgroep Twente (Almelo, The Netherlands).

### Study design and primary outcomes

#### Eforto® and muscle fatigability assessment

The Eforto® system comprises of the original large rubber bulb of the MV, connected to an analog Honeywell TruStability HSCMANN100PGAA3 gauge type pressure sensor, with signal axial barbed port, 0–100 PSI measurement range, and accuracy of ± 0.25% FSS BFSL (Full Scale Span Best Fit Straight Line). The sensor was connected to an analog-to-digital conversion (resolution = 10-bit and sample frequency = 100 Hz) and data transmission system based on the BITalino (r)evolution, which has been previously validated[20]. The system was connected via Bluetooth to the Eforto® application on a smartphone. Eforto® can be set to the professional or self-test modes. In the professional mode, a trained assessor provides the standardized instructions (same as MV) and encouragements during the test. The assessor can monitor the GS readings on the smartphone screen during the measurement. In the self-test mode, the participant is guided through the steps by the application providing standardized auditory and visual instructions and motivational cues. In both settings, GS readings are not visible to the participant during the measurement. Standardized instructions for the positioning of the bulb in the hand and fingers was provided for all test procedures, by the assessors (when using the MV and Eforto®) as well as by the smartphone app (both in the professional mode as in the self-test mode): bulb positioned in the middle of the hand with the tube of the MV or the Eforto® case pointing upwards, the four fingers along one side of the bulb and the thumb along the other side of the bulb.

Eforto® was calibrated prior to each assessment. The test protocol was based on previous studies conducted at VUB/UZB [8–10]. Briefly, participants were asked to maintain the following position: shoulder adducted and neutrally rotated, elbow flexed at 90°, forearm in neutral position, and wrist in slight extension (0°–30°). The GS_max_-test was repeated three times. Each time the participants squeezed the bulb as hard as possible with their dominant hand for five seconds. The highest score (in kPa) of the three attempts was noted as GS_max_. Next, for the FR-test, participants were instructed to squeeze the bulb as hard as possible and for as long as possible, until the GS dropped below 50% of its maximum. Standardized verbal encouragement (‘harder, harder’) was provided by the assessor (professional mode) or by the Eforto® application (self-test mode) each time the GS dropped by ≥ 2 kPa relative to the previous value. In both modes, the application verified the GS peak during the first five seconds of the FR-test and if the GS peak was less than 80% of the predetermined GS_max_, the test was aborted and restarted after at least 30 s of rest. In case the GS peak obtained during the FR-test exceeded the predetermined GS_max_, this score was set as the new GS_max_. The initial three GS_max_ measurements are required to obtain the maximum GS, which serves as the reference point for the FR test, which is a maximal endurance test. All data were automatically transferred to, and securely stored on, the online Eforto® platform.

#### Criterion validity

Sixty-one community-dwelling older persons performed the fatigability tests at the clinical study center once using the analog (MV) and once with Eforto® (professional mode). Tests were performed on the same day, in a random order with at least a 1-h interval. Each participant was tested by the same well-trained assessor (*n* = 4) for both measurements, except for four cases in which the assessor was changed between measurements due to unavailability. These four cases (2 women and 2 men) were excluded for the statistical analysis to prevent inter-assessor bias. Another case (1 man) was excluded from the statistical analysis due to technical problems leading to lost data on the Eforto® platform (Table 1).

#### Intra-rater reliability

Thirty community-dwelling older persons of the previous cohort performed the tests using Eforto® at home (self-test mode) without professional supervision. After an individual information session explaining how to use Eforto®, participants were asked to perform the test daily for one week starting from the first day after the measurements at the study center. For 28 participants, complete sets of measurements of six consecutive days were available and used for statistical analysis (Table 1).

#### Inter-rater reliability

Data from 29 participants of the same community-dwellers cohort were used to analyze inter-rater reliability by comparing the first home self-assessment with the measurement that took place at the clinical study center. One man had no study center data. In addition, 26 hospitalized geriatric patients and 25 patients with hip fracture completed two assessments (in professional mode) on the same day with at least a 1-h interval. Three cases were excluded (one geriatric patient and two patients with hip fracture, all women) due to data recording issues. One test was supervised by a researcher (*n* = 3) and one by a health care professional (*n* = 4). The test sequence depended on the availability of the health care professional (Table 1). In total 77 measurements were used to assess the inter-rater reliability.

### Secondary outcomes

#### Participants’ characteristics

Age, sex and living situation were noted for all participants. For the community-dwelling cohort, home medication was noted. For all participants Charlson’s Comorbidity Index [21] was computed.

### Statistical analysis

GW was calculated using two methods: the estimated GW (GW_estimated_) and actual measured GW (GW_measured_).

For the analog MV measure of GW, as the integral cannot be calculated we used the GW_estimated_ to evaluate the criterion validity, using the previously published equation [9–12, 22–26]:

1 $${\mathrm{GW}}_{\mathrm{estimated}}= 0.75*{\mathrm{GS}}_{\mathrm{max}}*FR$$

with GW_estimated_ being estimated grip work (kPa*s), GS_max_ being the highest maximal grip strength reached during GS_max_ and FR-test (kPa), FR being fatigue resistance (time (s) during which GS dropped to 50% of its maximum).

GW_measured_ was calculated by integrating the actual GS over time during the FR-test [10, 12, 26]:

2 $${\mathrm{GW}}_{\mathrm{measured}}= {\sum }_{(FR100-50)}\mathrm{GS}*t$$

With GW_measured_ is the measured grip work (kPa*s), GS is the grip strength (kPa), *t* is the time-interval (at 100 Hz = 0.01 s).

The difference in GS_max,_ and muscle fatigability (FR and GW_estimated_) measured with the analog MV and Eforto® was analyzed using a paired *t* test. Bland–Altman plots with limits of agreements and a linear regression line were plotted to verify proportional differences between both systems. Additionally, Pearson correlation coefficients were computed.

Intra-rater reliability of the GS_max,_ and muscle fatigability (FR and GW_measured_) were assessed using intra class correlations (ICC) derived from a 2-way mixed effects, absolute agreement, single measurement (measurements in self-test mode of six consecutive days) as well as a multiple measurements model (mean of two consecutive measurements during six days, i.e. mean day 1 + 2, day 3 + 4 and day 5 + 6) [27]. In addition, inter-rater reliability of the GW_measured_ was assessed using ICC, but derived from a 2-way random effects, absolute agreement, single rater/measurement model [27]. Finally, standard error of measurement (SEM_agreement_ = √(variance in repetition + measurement error variance)), smallest detectable change (SDC = 1.96*$\surd 2$*SEM_agreement_), systematic error proportion (SEP = variance in repetition/(subject variance + variance in repetition + measurement error)), and residual error proportion (REP = measurement error/(subject variance + variance in repetition + measurement error)) were calculated [28] (Table 1).

**Fig. 2:**
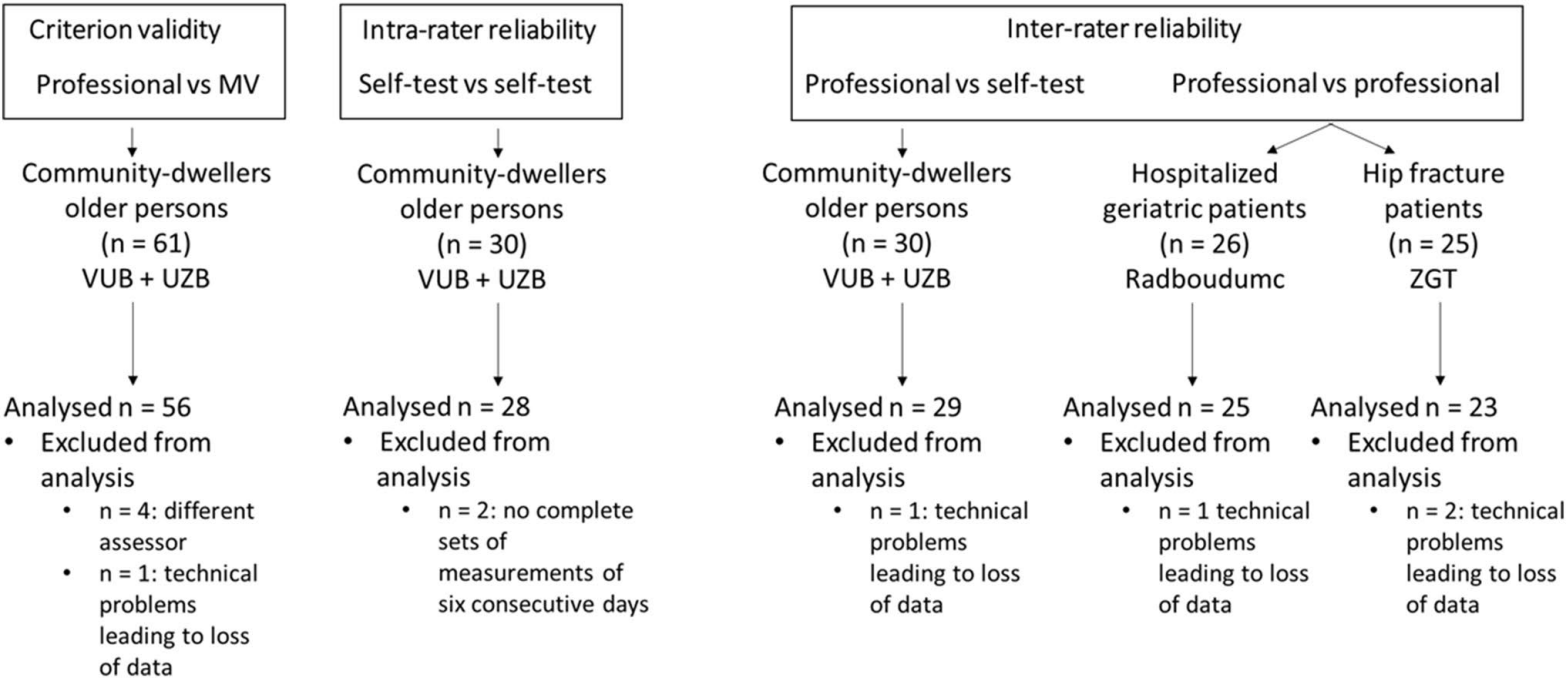
Study flow chart. *MV* Martin Vigorimeter, *VUB—UZB* Vrije Universiteit Brussel & Universitair Ziekenhuis Brussel, Belgium, *Radboud* Radboud University Medical Center, The Netherlands, *ZGT* Ziekenhuis Groep Twente, The Netherlands

For interpretation of the ICC-values, the following criteria were used: 0.60 ≤ ICC < 0.70 = moderate, 0.70 ≤ ICC < 0.80 = good, 0.80 ≤ ICC < 0.90 = very good, and ICC ≥ 0.90 = excellent [8]. Significance was set a priori at *p* < 0.05 (Fig. 2).

## Results

Participants’ characteristics are presented in Table 1. In brief, 61 community-dwelling old persons (mean age = 85.6 ± 3.3 years), 26 hospitalized geriatric patients and 25 patients with hip fracture participated (mean age = 84.1 ± 6.4 years and 81.9 ± 7.0 years).

### Criterion validity compared to MV system

**Fig. 3:**
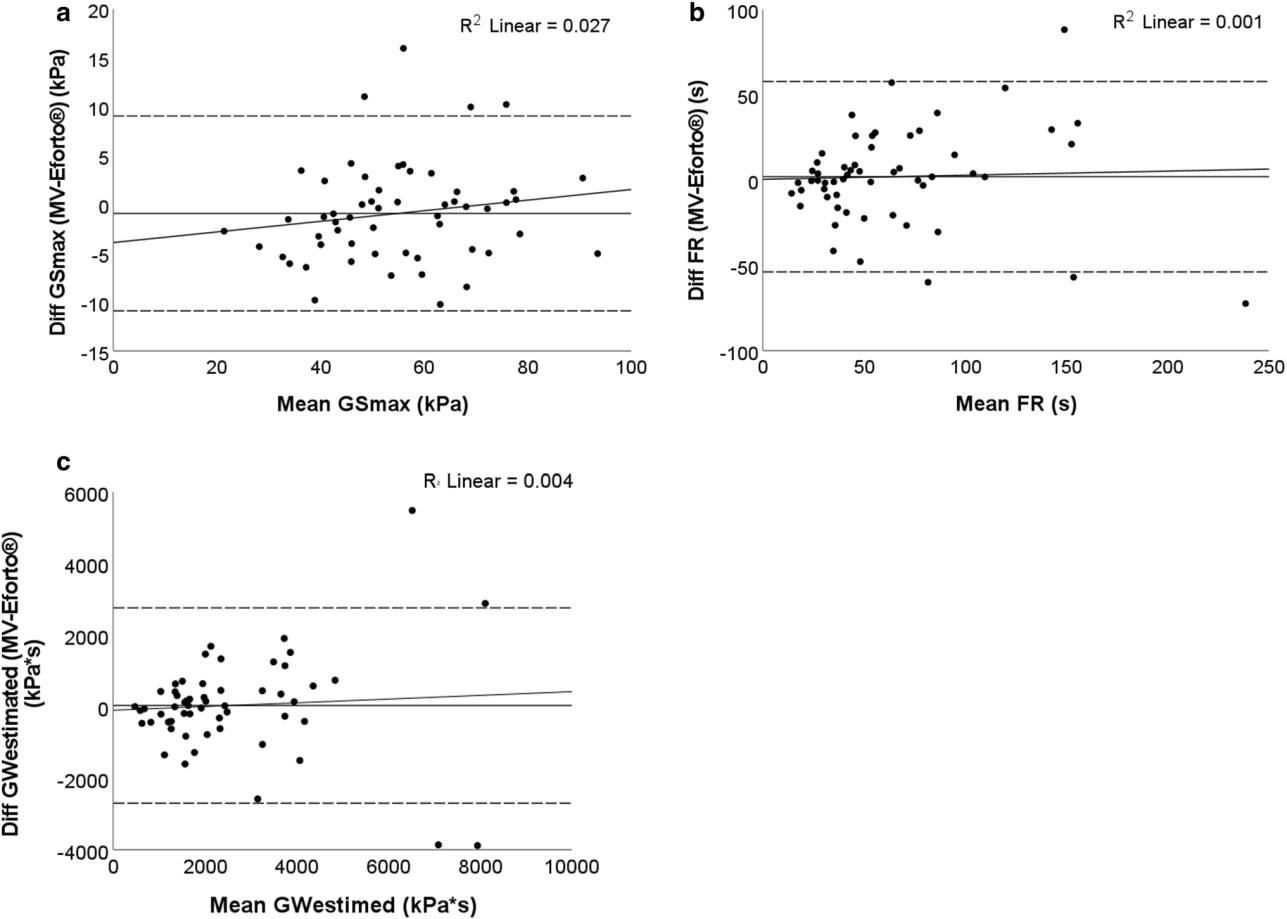
Bland–Altman plots for GS_max_ and muscle fatigability measured with MV and Eforto®. Presented data are derived from the community-dwelling older persons who performed the grip strength tests at the clinical study center twice using the analog (MV) and Eforto® (professional mode) (*n* = 56). The horizontal dotted lines show the upper and lower limits of agreement. The horizontal plain line represents the mean difference in respectively GS_max_, FR and GW_estimated_ between both systems. The other plain line represents the linear regression showing that there is no significantly proportional difference in GS_max_, FR and GW_estimated_ measured with both systems (GS_max_ *R*^2^ = 0.027, *p* = 0.223; FR *R*^2^ = 0.001, *p* = 0.786; GW_estimated_ *R*^2^ = 0.004, *p* = 0.633). In the first figure the horizontal axis represents the mean of GS_max_ and the vertical axis represents the difference between GS_max_ measured with MV and Eforto® device. In the second figure the horizontal axis represents the mean of FR and the vertical axis represents the difference between FR measured with MV and Eforto® device. In the third figure the horizontal axis represents the mean of GW and the vertical axis represents the difference between GW measured with MV and Eforto® device

As shown in Table 2, no significant differences were found between MV and Eforto®, neither in GS_max_ nor in muscle fatigability (FR and GW_estimated_). Moreover, MV and Eforto® showed good to excellent correlations for the three outcomes (ranging from 0.67 to 0.95, Table 3). Correlations were generally the highest for GS and lowest for GW_estimated_. Bland–Altman plots (shown in Fig. 3) showed no evidence for a proportional difference in GS_max_ or muscle fatigability between both systems. Similar results were obtained when analyzing male and female separately (shown in Table 2 and Supplementary Fig. 1).

**Table 2:** GS_max_, FR and GW_estimated_ measured with an analog and Eforto® system in community-dwelling persons aged 80 +

Population	Muscle fatigability parameters	MV	Eforto®	Mean difference ± standard error [95% confidence interval of the difference]	*p* value*
All (*n* = 56)	GS_max_ (kPa)	54.5 ± 16.1	55.4 ± 15.3	− 0.9 ± 0.7 [− 2.3–0.4]	0.179
	FR (s)	65.3 ± 46.9	63.2 ± 45.9	2.0 ± 3.8 [− 5.6–9.7]	0.595
	GW_estimated_ (kPa*s)	2567.9 ± 1924.2	2533.8 ± 1839.8	34.0 ± 186.4 [− 339.5–407.6]	0.856
Women (*n* = 32)	GS_max_ (kPa)	46.3 ± 12.1	47.4 ± 11.5	− 1.1 ± 1.0 [− 3.0–0.9]	0.267
	FR (s)	66.2 ± 48.7	60.7 ± 38.5	4.5 ± 5.4 [− 6.6–15.5]	0.417
	GW_estimated_ (kPa*s)	2222.9 ± 1828.1	2182.8 ± 1720.5	40.2 ± 254.4 [− 478.7–559.0]	0.876
Men (*n* = 24)	GS_max_ (kPa)	65.4 ± 14.3	66.1 ± 13.1	− 0.7 ± 1.0 [− 2.7–1.3]	0.463
	FR (s)	64.0 ± 45.5	65.2 ± 54.4	− 1.2 ± 5.3 [− 12.0–9.7]	0.825
	GW_estimated_ (kPa*s)	3027.8 ± 1990.9	3001.9 ± 1924.7	25.8 ± 278.5 [− 550.3–601.9]	0.927

*Paired sample *t* test; Values are expressed as mean ± standard deviation

*GS*_*max*_ maximal grip strength, *FR* fatigue resistance, *GW*_*estimated*_ grip work estimated (= Eq. 1), *MV* Martin Vigorimeter

**Table 3:** Correlations between analog handgrip (MV) and Eforto® system for GS_max_, FR and GW_estimated_ in community-dwelling persons aged 80+

	GS_max_ Eforto®			FR Eforto®			GW_estimated_ Eforto®		
	Overall (*n* = 56)	Women (*n* = 32)	Men (*n* = 24)	Overall (*n* = 56)	Women (*n* = 32)	Men (*n* = 24)	Overall (*n* = 56)	Women (*n* = 32)	Men (*n* = 24)
GS_max_ MV	*r* = 0.95	*r* = 0.90	*r* = 0.94						
FR MV				*r* = 0.81	*r* = 0.78	*r* = 0.88			
GW_estimated_ MV							*r* = 0.73	*r* = 0.67	*r* = 0.76

*r* = Pearson correlation coefficient

*GS*_*max*_ maximal grip strength, *FR* fatigue resistance, *GW*_*estimated*_ grip work estimated (= Eq. 1), *MV* Martin Vigorimeter. All correlation coefficients are statistically significant at *p* < 0.001

### Intra-rater reliability

Self-assessment of GW_measured_ on six consecutive days showed good reliability in community-dwellers (ICC = 0.73 [0.61–0.85]); which improved to excellent (ICC = 0.94 [0.89–0.97]) when the average values of two consecutive days were considered. The relatively small SEM_agreement_ indicates minimal random and systematic error (Table 4).

**Table 4:** Intra-rater and inter-rater reliability of the GW_measured_

Population	ICC [95% confidence interval]	SEP	REP	SEM_agreement_ (kPa*s)	SDC (kPa*s)
Intra-rater reliability					
Community-dwelling older persons (*n* = 28)	0.73 [0.61–0.85]^a^	0.004	0.263	699.5	1938.78
	0.94 [0.89–0.97]^b^	0.005	0.150	496.5	1376.36
Inter-rater reliability					
Community-dwelling older persons (*n* = 29)	0.59 [0.29–0.78]	0.002	0.413	661.5	1833.58
Hospitalized geriatric patients (*n* = 25)	0.86 [0.72–0.94]	0.004	0.132	224.5	622.15
Patients with hip fracture (*n* = 23)	0.79 [0.58–0.91]	0.000	0.205	386.5	1071.22

*ICC* intra class correlation coefficient, ^a^single measurement model (six consecutive days), ^b^multiple measurements model (average of day1 + day2, day3 + day4 and day5 + day6), *SEP* systematic error proportion, *REP* residual error proportion, *SEM*_*agreement*_ standard error of measurement, *SDC* smallest detectable change

Note, the SEM should be interpreted as follows: Larger values indicate lower reliability. If reliability (ICC) = 0, the SEM will be equal to the standard deviation (SD) of the observed test scores. If the ICC = 1, the SEM will be zero. For example, the SD of the grip work measurements in the community-dwelling sample was 1839.78 (Table 2). The SEM for the intra-rater reliability (single measurement) in this group was 699.5 with an ICC of 0.73. As the SEM is much lower than the SD, the SEM can be considered as relatively small

### Inter-rater reliability

Inter-rater reliability of GW_measured_ showed good to very good ICC-values for both hospitalized geriatric patients and patients with hip fracture (ICC = 0.86[0.72–0.94] and 0.79[0.58–0.91]), whereas the ICC was moderate for the community-dwellers (ICC = 0.59[0.29–0.78]) (Table 4). SEM_agreement_ values were small in geriatric inpatients and patients with hip fracture (respectively 224.5 and 386.5 kPa*s), indicating minimal random and systematic error, but greater in community-dwellers (661.5 kPa*s). FR-values followed the same trend as GW_measured_ while GS_max_ was not that different between the three populations. See Supplementary Table 1 and 2 for intra- and inter-rater reliability analysis of GS_max_ and FR.

## Discussion

The first objective of this study was to evaluate the criterion validity of Eforto® to measure muscle fatigability using the classic analog MV measure as the reference standard. The good to excellent correlation between the two instruments in GS_max_ and muscle fatigability confirms the validity of Eforto® in community-dwelling older persons. The correlation between the instruments for GS_max_ was higher than for FR. This difference in correlation is explained by the fact that the GS_max_-test was performed four times (three consecutive GS_max_ attempts followed by a fourth effort during the FR test), while the FR-test was performed only once. Hence, the variation in repeated GS_max_ values is reduced by averaging over the four attempts. Previous evidence showed that the evaluation of sustained voluntary contractions is more reproducible after earlier familiarization with maximal voluntary contractions [29]. On the other hand, we cannot exclude the possibility that Eforto® may measure FR more accurately than the analog method, because the data registration starts and stops automatically based on the strength increase and subsequent strength decay as programmed in the application. Conversely, when assessing FR with the analog MV, the time from the start of the test until GS dropped to 50% of its maximum is measured by the assessor using a handheld stopwatch, which may be less accurate than the Eforto® application. Consequently, the correlation between Eforto® and MV for GW_estimated_ is smaller than that for FR. Nevertheless, the small and non-significant differences between the two systems for GS_max_ and muscle fatigability indicate the absence of a systematic measurement error.

The second aim of this study was to examine the inter-rater and intra-rater reliability of the GW_measured_ results obtained with Eforto®. Both inter-rater and intra-rater reliability of GW_measured_ were moderate to excellent, with ICC-values ranging from 0.59 to 0.94. This is in line with a previous study that reported good to excellent inter- and intra-assessor reliability ICC-values of FR obtained with the analog MV in hospitalized geriatric patients ranging from 0.77 to 0.94 [8]. Moreover, we found that intra-rater reliability of GW_measured_ improved when using the average of the values of two consecutive days compared to the values of six consecutive days, thus it seems that for long term monitoring, two measurements per week are sufficient. But it could also mean that there is substantial day-to-day variation. Further research should clarify if the day-to-day variation gives clinically relevant information or not. Additionally, inter-rater reliability ICC-values for muscle fatigability in old community-dwellers are lower compared to the other two population groups, whereas the ICC-values for GS_max_ are less different between the three populations. Moreover, the SEM-values for FR and GW_measured_ were higher for community-dwellers than the hospitalized populations. This might be due to differences in test modes of Eforto® that were used (professional mode in the hospitals versus self-test mode in the community-dwellers). Also, the repeated tests were performed on the same day in the hospitals and on consecutive days in the community-dwellers cohort. Finally, our results show that the ICC-values for GS_max_ are high but those of FR remain lower, probably because of the bigger residual error. In future studies, the error variance could likely be reduced by shortening the FR-test protocol (i.e., until 25% drop instead of until 50% drop in GS_max_) as suggested in a previous study[26].

According to the WHO, “fostering Healthy Ageing will require a much better understanding of common trajectories of intrinsic capacity and functional ability, their determinants and the effectiveness of interventions to modify them”. This is exactly what the Eforto® system aims to do: to offer a method for measuring and monitoring the intrinsic capacity [30, 31] of a person. Unique to Eforto® is that rather than focusing on a statistic measure (GS), it utilizes information from the dynamic performance of the individual (GW) to recruit or actualize that potential. This is more sensitive to identify early decline in intrinsic capacity[32] and enables to detect changes in intrinsic capacity long before frailty or dependency are detected by other methods. GW opens a window of opportunity for preventive measures, e.g., diet [33] and exercise [34, 35], to optimize the level of intrinsic capacity before the decline becomes problematic.

A limitation of this study is that we did not collect data on body composition including presence of sarcopenia. The exclusion criteria may result in a somewhat selected sample, which could limit generalizability of the findings. However, the reliability was evaluated in three distinct patient groups, with ICCs being in the same range for hospitalized geriatric patients and patients with hip fracture. In this study we used pressure readouts to measure GS, FR and GW since the pressure measured in the bulb reflects the muscle strength produced by the participant. However, Pneumatic systems, such as rubber bulbs, are better tools to measure muscle fatigability and more valid to discriminate between subjects of varying degrees of frailty compared with clamp-based instruments such as the Jamar dynamometer [11, 12]. In addition, it has been previously shown that GS measured by the Jamar dynamometer is even more dependent on hand anthropometry than measurement with the Martin Vigorimeter which is a Pneumatic device with exactly the same rubber bulb as used in Eforto® [36]. Finally, standardized instructions for the positioning of the bulb in the hand and fingers was provided for all test procedures, by the assessors (when using the Martin Vigorimeter and Eforto®) as well as by the smartphone app (both in the professional mode as in the self-test mode).

A strength is that validity and reliability were evaluated in three distinct clinically relevant settings, which demonstrates the clinimetrics of Eforto in every day clinical practice.

## Conclusion

Overall, we conclude that Eforto® is a valid and reliable tool to measure muscle fatigability, which can be used in older persons both in supervised clinical settings as well as in unsupervised community settings.

## Supplementary Information

Below is the link to the electronic supplementary material.

Supplementary file1 (TIF 96 KB)

Supplementary file2 (TIF 92 KB)

Supplementary file3 (TIF 101 KB)

Supplementary file4 (PDF 100 KB)

## Data Availability

All data generated or analyzed during this study are included in this article or its supplementary material files. Further enquiries can be directed to the corresponding authors.
