# Supplementary figures and images for: Validity and reliability of Eforto®, a system to (self-)monitor grip strength and muscle fatigability in older persons
Source: Aging Clin Exp Res. 2023 Mar 10;35(4):835–45. doi: 10.1007/s40520-023-02365-3 (PMC10115702; doi:10.1007/s40520-023-02365-3)

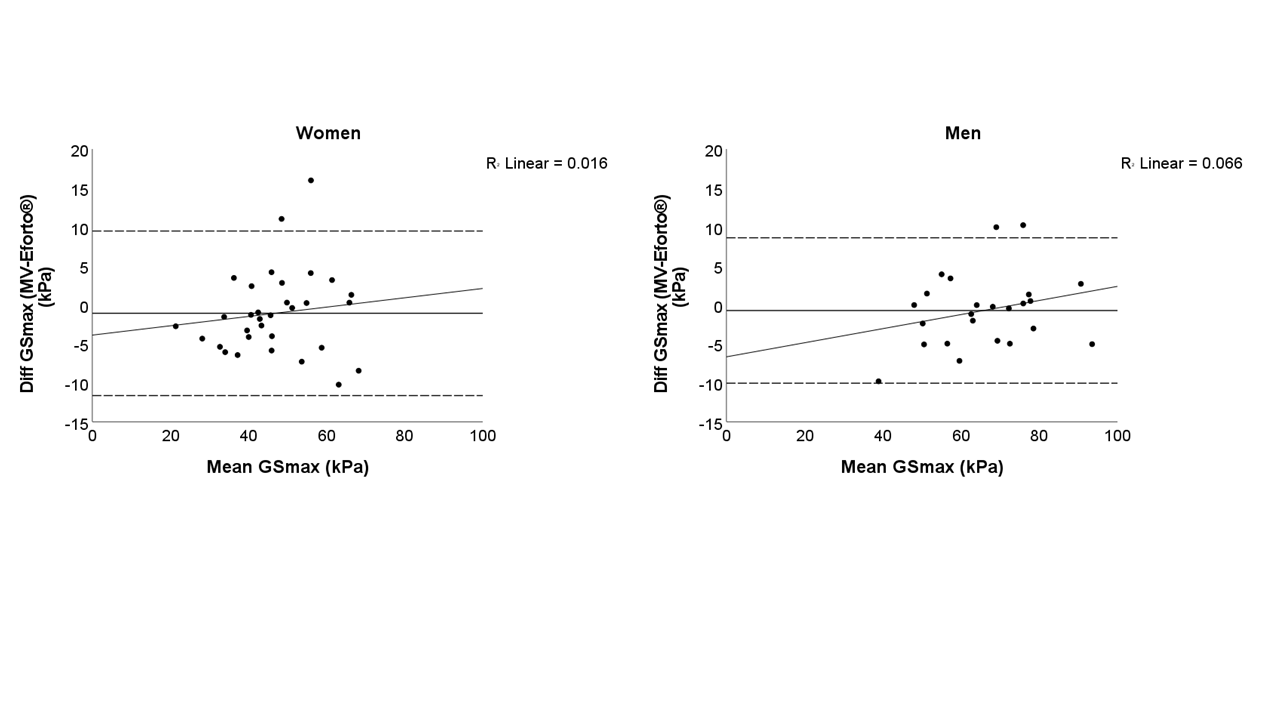

Supplement: Supplementary file 1 — Supplementary file1 (TIF 96 KB) [file 40520_2023_2365_MOESM1_ESM.tif]

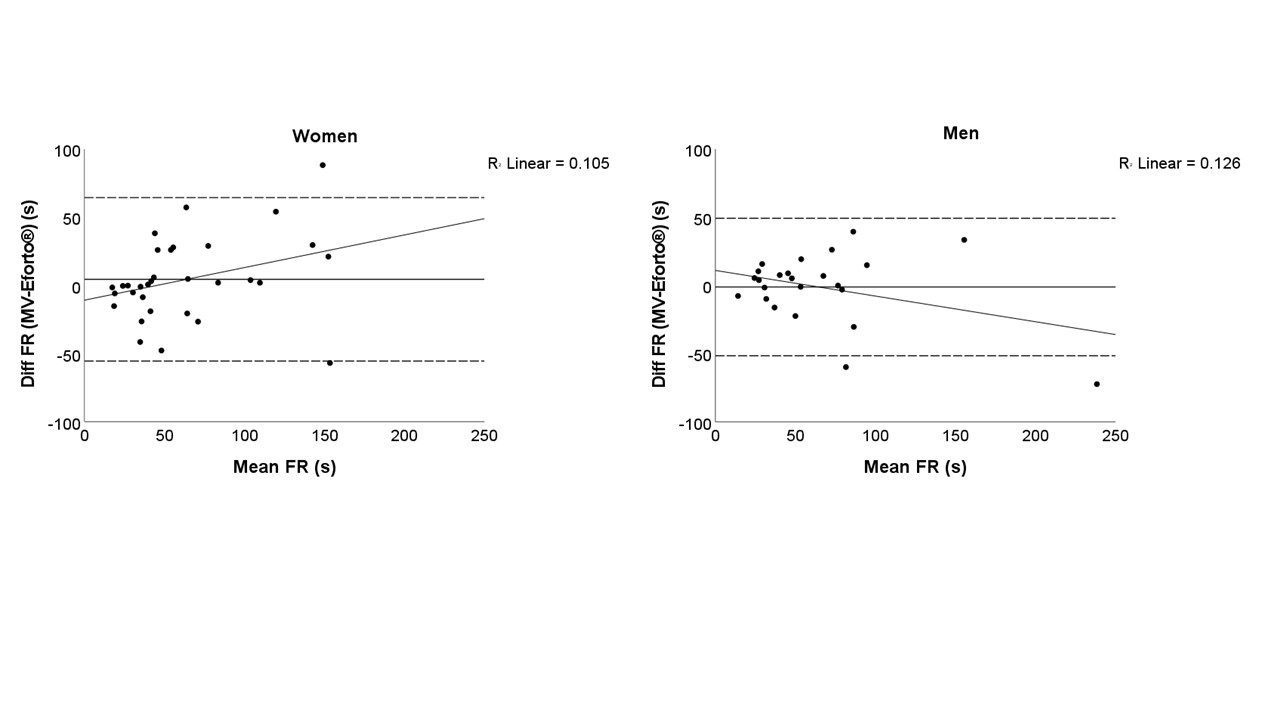

Supplement: Supplementary file 2 — Supplementary file2 (TIF 92 KB) [file 40520_2023_2365_MOESM2_ESM.tif]

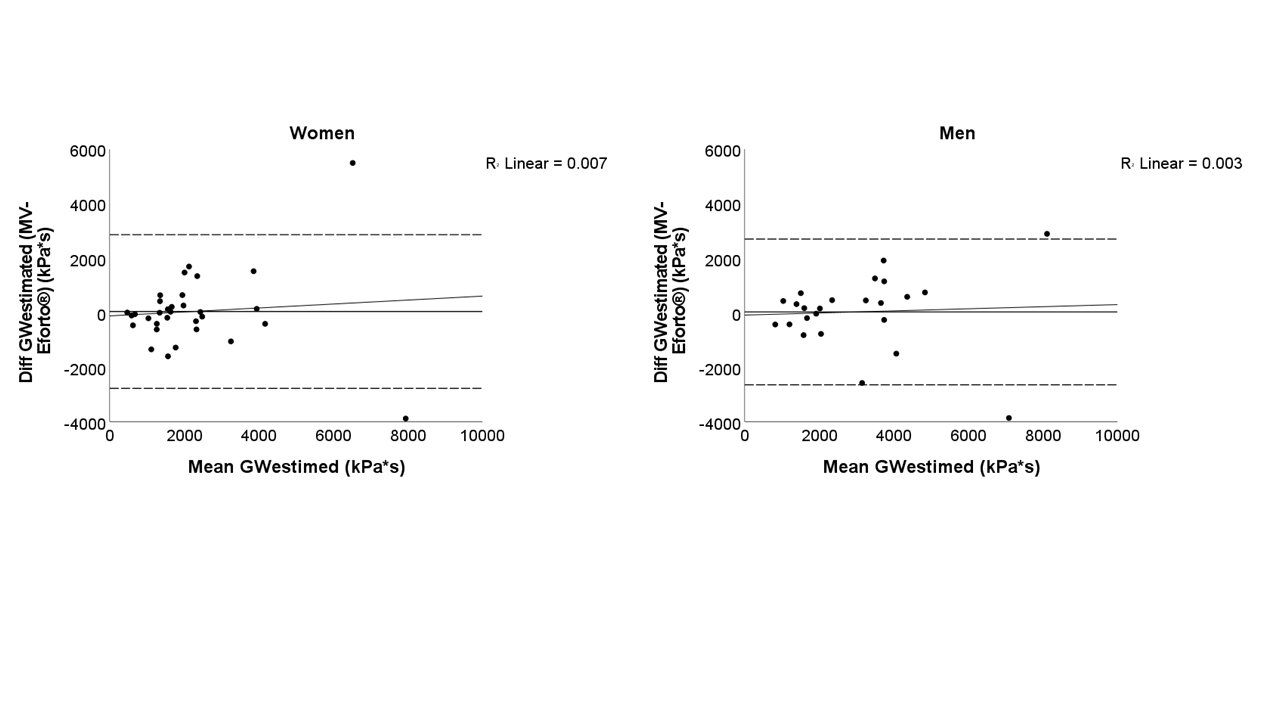

Supplement: Supplementary file 3 — Supplementary file3 (TIF 101 KB) [file 40520_2023_2365_MOESM3_ESM.tif]
