# Supplementary material for: Validity and reliability of Eforto®, a system to (self-)monitor grip strength and muscle fatigability in older persons
Source: Aging Clin Exp Res. 2023 Mar 10;35(4):835–45. doi: 10.1007/s40520-023-02365-3 (PMC10115702; doi:10.1007/s40520-023-02365-3)
Supplement: Supplementary file 4 — Supplementary file4 (PDF 100 KB) [file 40520_2023_2365_MOESM4_ESM.pdf]

VALIDITY AND RELIABILITY OF EFORTO®, A SYSTEM TO (SELF-)MONITOR GRIP STRENGTH AND MUSCLE FATIGABILITY IN OLDER PERSONS.

1. SUPPLEMENTARY MATERIAL

Supplementary Table 1: Intra-rater and inter-rater reliability of GS_{max}

Population	ICC [95% confidence interval]	SEP	REP	$SEM_{agreement}$ (kPa)	SDC (kPa)
Intra-rater reliability					
Community-dwelling older persons (n=28)	0.94 [0.90-0.97][a]	0.000	0.063	4.32	11.97
	0.99 [0.98-0.99][b]	0.000	0.036	3.22	8.92
Inter-rater reliability					
Community-dwelling older persons (n=29)	0.89 [0.78-0.95]	0.000	0.109	5.24	14.53
Hospitalized geriatric patients (n=25)	0.91 [0.82-0.96]	0.003	0.084	4.90	13.58
Hip fracture patients (n=23)	0.90 [0.79-0.96]	0.000	0.096	3.34	9.26

ICC = intra class correlation coefficient, [a]single measurement model (six consecutive days), [b]multiple measurements model (average of day1+day2, day3+day4 and day5+day6), SEP = systematic error proportion, REP = residual error proportion, $SEM_{agreement}$ = standard error of measurement, SDC = smallest detectable change.

Supplementary Table 2: Intra-rater and inter-rater reliability of FR

Population	ICC [95% confidence interval]	SEP	REP	$SEM_{agreement}$ (s)	SDC (s)
Intra-rater reliability					
Community-dwelling older persons (n=28)	0.64 [0.49-0.78][a]	0.001	0.363	18.30	50.72
	0.93 [0.86-0.96][b]	0.005	0.190	11.98	33.21
Inter-rater reliability					
Community-dwelling older persons (n=29)	0.43 [0.09-0.68]	0.003	0.568	19.20	53.21
Hospitalized geriatric patients (n=25)	0.66 [0.38-0.84]	0.006	0.330	9.10	25.23
Hip fracture patients (n=23)	0.85 [0.69-0.93]	0.001	0.146	14.29	39.62

ICC = intra class correlation coefficient, [a]single measurement model (six consecutive days), [b]multiple measurements model (average of day1+day2, day3+day4 and day5+day6), SEP = systematic error proportion, REP = residual error proportion, $SEM_{agreement}$ = standard error of measurement, SDC = smallest detectable change.

Supplementary Figure 1: Bland-Altman plots for GS_{max} and muscle fatigability measured with MV and Eforto®, per sex

Presented data are derived from the community-dwelling older persons who performed the grip strength tests at the clinical study center twice using the analog (MV) and Eforto® (professional mode) (women n=32 and men n=24). The horizontal dotted lines show the upper and lower limits of agreement. The horizontal plain line represents the mean difference in respectively GS_{max}, FR and $GW_{estimated}$ between both systems. The other plain line represents the linear regression showing that there is no significant proportional difference in GS_{max}, FR and $GW_{estimated}$ measured with both systems when looking for sex separately (GS_{max} women $R^2 = 0.016$, $p = 0.487$ and men $R^2 = 0.066$, $p = 0.225$; FR women $R^2 = 0.105$, $p = 0.070$ and men $R^2 = 0.126$, $p = 0.089$; $GW_{estimated}$ women $R^2 = 0.007$, $p = 0.656$ and men $R^2 = 0.003$, $p = 0.810$). In the first figure the horizontal axis represents the mean of GSmax and the vertical axis represents the difference between GSmax measured with MV and Eforto® device. In the second figure the horizontal axis represents the mean of FR and the vertical axis represents the difference between FR measured with MV and Eforto® device. In the third figure the horizontal axis represents the mean of GW and the vertical axis represents the difference between GW measured with MV and Eforto® device.
